# Divergent self-assembly dynamics of squalene-derived bolaamphiphiles enabled by ethylene glycol-length-switchable Ostwald ripening

**DOI:** 10.1039/d6ra03150a

**Published:** 2026-07-03

**Authors:** Tran Ngoc Linh, Rintarou Ootani, Hirohmi Watanabe, Takashi Arimura, Hiroko Isoda, Masato Kawasaki

**Affiliations:** a Research Institute for Sustainable Chemistry, National Institute of Advanced Industrial Science and Technology (AIST) 3-11-32, Kagamiyama Higashihiroshima Hiroshima 739-0046 Japan; b Open Innovation Laboratory for Food and Medicinal Resource Engineering (FoodMed-OIL), National Institute of Advanced Industrial Science and Technology (AIST), Laboratory of Advanced Research D, University of Tsukuba Tsukuba Ibaraki 305-0006 Japan takashi-arimura@aist.go.jp; c Institute of Life and Environmental Sciences, University of Tsukuba Tsukuba Ibaraki 305-8572 Japan; d Institute of Materials Structure Science, Inter-University Research Institute Corporation, High Energy Accelerator Research Organization (KEK) 1-1 Oho Tsukuba Ibaraki 305-0801 Japan

## Abstract

Bolaamphiphiles were synthesized by introducing ethylene glycol groups at both terminal double bonds of squalene for the first time. The monolayer vesicle structures formed in aqueous media were elucidated by liquid cell and cryogenic transmission electron microscopy. The morphology of the self-assembled aggregates was found to be strongly dependent on the length of the ethylene glycol group. Notably, bolaamphiphiles bearing two tetra-ethylene glycol groups formed exceptionally ultra-small monolayer vesicles with an average diameter of 10 nm, which exhibited remarkable stability for a period of 180 days. Substitution with tri-ethylene glycol groups preserves the monolayer vesicular architecture while promoting a gradual size increase from 160 nm to 1600 nm over 180 days. The growth of particle diameter was elucidated to proceed *via* an Ostwald ripening-driven mechanism under diffusion-limited conditions. During the vesicle growth process, a biphasic pathway featuring two distinct diffusion coefficients was identified within the framework of the Lifshitz–Slyozov–Wagner theory, and we propose, to our knowledge, a mechanistic model in which each phase is governed by different driving forces.

## Abstract 2

Bola-triEGSQ vesicles grow from 160 to 1600 nm over 180 days, consistent with Ostwald ripening, while tetraEG analogues remain unchanged.

## Graphical abstract

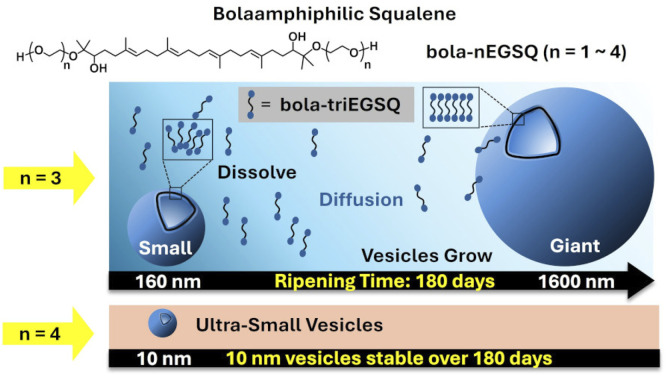

## Introduction

Amphiphiles attract significant interest in nanoscience and medicine due to their ability to self-assemble into soft matter structures such as micelles, vesicles, and emulsions. In particular, molecules possessing two hydrophilic end groups connected by a single hydrophobic group are termed bolaamphiphiles and form monolayer lipid membranes (MLMs).^1^ The self-assembled structures formed by MLMs exhibit significantly higher stability compared to those formed by phospholipid bilayers composed of amphiphilic molecules possessing a single hydrophilic group.^2^ In fact, archaebacterial lipids found in extreme environments such as high temperatures, high salt concentrations, and anaerobic conditions are composed of bolaamphiphiles, like archaeosomes, and are being applied to biomedical fields such as drug delivery systems (DDS).^3–5^ However, the isolation and extraction of bolaamphiphiles from natural resources are extremely difficult, so significant interest has been focused on chemical synthesis.^5^ Currently, self-assembled structures generated from various bolaamphiphiles are being actively researched for practical applications in nanomaterials research^6–12^ and, furthermore, in DDS^13–16^ for the treatment of intractable diseases.^17^

We have already shown that amphiphilic molecules, in which ethylene glycol (EG) groups are selectively introduced at one of the terminal double bond of squalene, self-assemble into bilayer-structured vesicles.^18,19^ Furthermore, it was discovered for the first time that these compounds exhibit physiological activities varying with the length of the EG group, including bladder cancer suppression,^20^ malignant melanoma metastasis reduction,^21^ and hair growth-promoting effects.^22^ During this study, we found that bolaamphiphiles derived from squalene form stable self-assembled structures with MLMs architecture, showing potential for practical applications. Notably, the synthesized bolaamphiphiles themselves were found to be stable in solution even at elevated temperatures (*e.g.*, 100 °C). However, the stability of their vesicular assemblies under extreme conditions, such as high temperatures and high salt concentrations, has not yet been systematically investigated and remains an important issue.

In this study, we first synthesized bolaamphiphiles (bola-nEGSQ) in which nEG (*n* = 1–4) groups were introduced at both terminal double bonds of squalene. Using liquid cell transmission electron microscopy (LC-TEM) and cryogenic transmission electron microscopy (Cryo-TEM) observations, we conducted a detailed investigation into the dependence of the self-assembled structures of bola-nEGSQ on EG chain length. In particular, it was elucidated that the vesicles formed by bola-triEGSQ exhibit the Ostwald ripening effect, undergoing gradual growth over a period of six months.

## Results and discussion

**Scheme 1:**
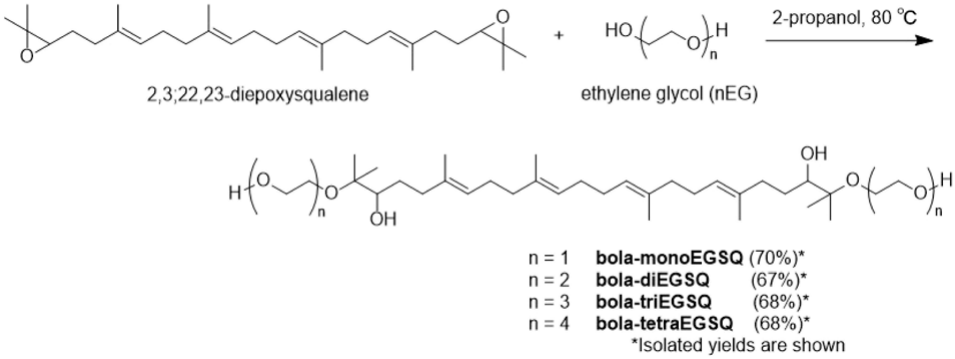
Preparation of bolaamphiphilic squalene bearing ethylene glycol groups (bola-nEGSQ) from 2,3;22,23-diepoxysqualene.

As shown in Scheme 1, four bolaamphiphilic squalene molecules were synthesized for the first time through the reaction of 2,3;22,23-diepoxysqualene^23,24^ and the corresponding ethylene glycol. Details of the synthetic procedures are provided in the SI. The purities of the bolaamphiphiles, as determined by quantitative NMR, are greater than 95%. The present transformation is proposed to involve weak activation of the epoxide through hydrogen bonding with the alcohol, which may generate a small degree of cationic character consistent with a pathway with partial S_*N*_1-like character. Consequently, regioselective ring opening at the more substituted carbon is expected to be favoured. As the reaction requires heating at 80 °C and thus involves a relatively high activation barrier, differences in steric demand and nucleophilicity are likely to become more pronounced under these kinetically slow conditions. On this basis, primary alcohols may react more readily than the more sterically hindered 2-propanol.

**Fig. 1:**
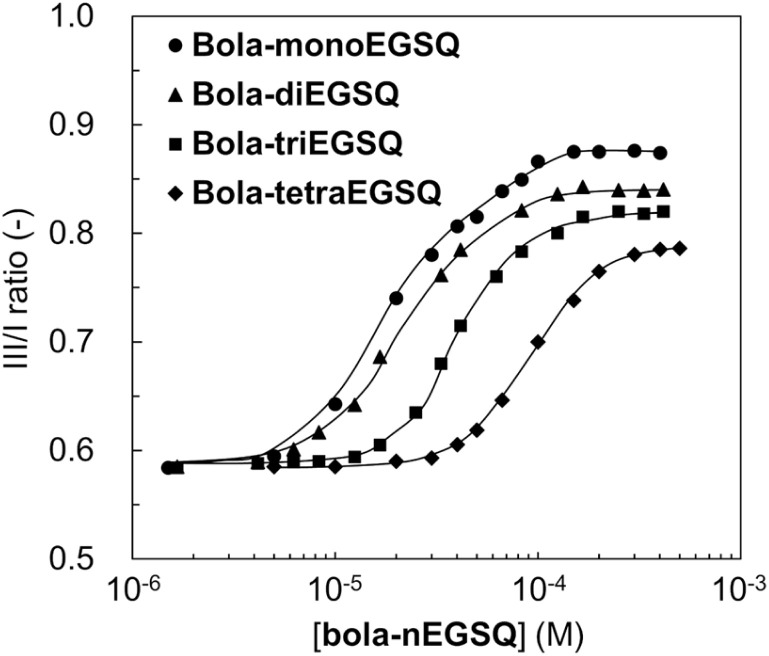
Plot of the III/I ratio in pyrene fluorescence *versus* the concentration of bola-nEGSQ in water, 25 °C at 1 h post-mixing; [pyrene] = 5.0 × 10^−7^ M, excitation at 335 nm.

The vibronic band intensities in the pyrene monomer fluorescence are frequently used as a probe to determine the critical aggregation concentration (CAC) values of micelles and vesicles.^25^ As shown in Fig. 1, we found that the intensity ratio of band III to band I (III/I ratio) of pyrene in water increases with increasing the bola-nEGSQ concentration and reaches a saturation value (0.82–0.87) above 1.1 × 10^−4^ M for bola-monoEGSQ, bola-diEGSQ, and bola-triEGSQ at 1 h post-mixing. In the case of bola-tetraEGSQ, the saturation value was 0.78.

Such a finding is consistent with bola-nEGSQ being organized in a hydrophobic domain in water. A plot of III/I ratio for bola-nEGSQ of this study gave the continuous saturation curvature (sigmoid function), leading us to suggest that the CAC values are not so sharp as they are for sodium dodecyl sulfate.^25^ Thus, the CAC values were estimated as being 1.2 × 10^−4^ M for bola-monoEGSQ, 1.3 × 10^−4^ M for bola-diEGSQ, 2.0 × 10^−4^ M for bola-triEGSQ, and 3.5 × 10^−4^ M for bola-tetraEGSQ.

Careful examination of Fig. 1 reveals that the curvatures for bola-monoEGSQ and bola-diEGSQ start rising at approximately 8.0 × 10^−6^ M, whereas those of bola-triEGSQ and bola-tetraEGSQ rise at 2.0 × 10^−5^ M and 4.0 × 10^−5^ M, respectively. This indicates that bola-triEGSQ and bola-tetraEGSQ are highly soluble as monomers in aqueous solution and are unlikely to incorporate pyrene.

The aggregation sizes of bola-nEGSQ determined by dynamic light scattering (DLS), and the corresponding zeta potentials are summarized in Table 1. Measurements were conducted at concentrations slightly below the CAC and at 1 h post-mixing. Hydrodynamic diameters of bola-monoEGSQ, bola-diEGSQ, and bola-triEGSQ ranged from 147 to 194 nm, whereas bola-tetraEGSQ formed significantly smaller aggregates, approximately 10 nm in diameter. The polydispersity index (PDI) was approximately 0.1, indicating a nearly monodisperse particle distribution. Interestingly, at a concentration of 1.0 × 10^−5^ M, either bola-triEGSQ nor bola-tetraEGSQ exhibited detectable aggregation by DLS, consistent with their solubilization as monomers.

**Table 1:** Hydrodynamic diameter (*D*) determined by DLS, polydispersity index (PDI) and zeta potential (*ζ*) of bola-nEGSQ assembles in water, 25 °C at 1 h post-mixing

Compounds	Conc. (M)	*D* a (nm)	PDI^a^ (−)	*ζ* a (mV)
Bola-monoEGSQ	1.0 × 10^−4^	194 ± 2	0.15 ± 0.02	−31 ± 4
Bola-diEGSQ	1.0 × 10^−4^	147 ± 4	0.12 ± 0.01	−33 ± 3
Bola-triEGSQ	1.0 × 10^−4^	193 ± 8	0.07 ± 0.02	−27 ± 2
Bola-tetraEGSQ	5.0 × 10^−4^	10 ± 2	0.17 ± 0.02	−15 ± 1

a Values are expressed as mean ± SD of at least three independent measurements.

**Fig. 2:**
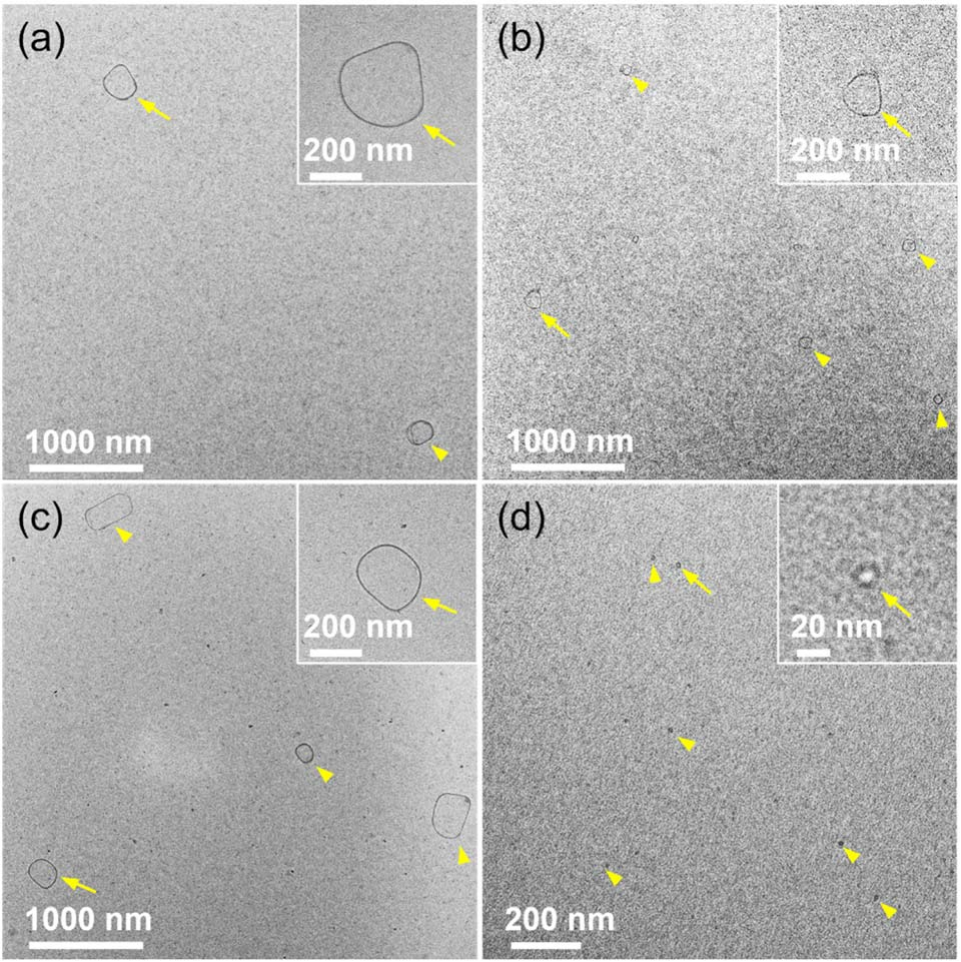
LC-TEM images of (a) bola-monoEGSQ, (b) bola-diEGSQ, (c) bola-triEGSQ, and (d) bola-tetraEGSQ assembles in water, 25 °C at 1 h post-mixing; [bola-nEGSQ] = 1.0 × 10^−4^ M.

**Fig. 3:**
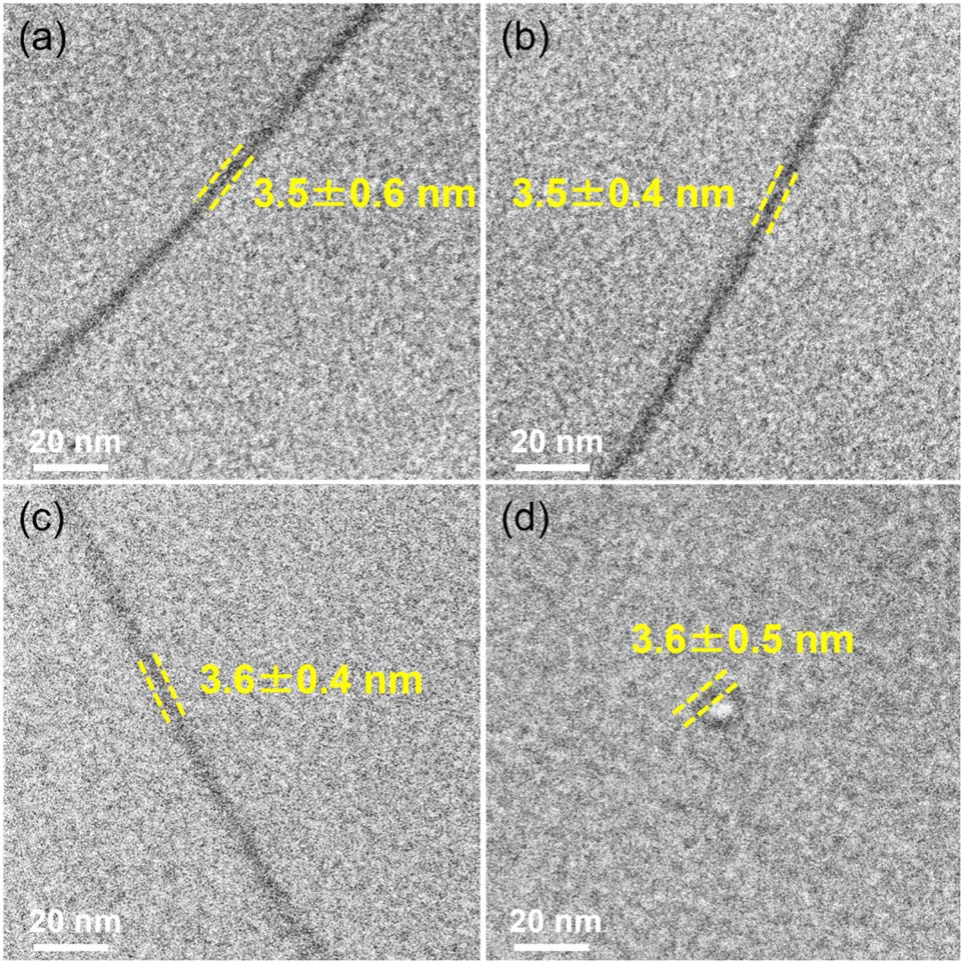
LC-TEM images of layer thickness of (a) bola-monoEGSQ, (b) bola-diEGSQ, (c) bola-triEGSQ, and (d) bola-tetraEGSQ vesicles in water, 25 °C at 1 h post-mixing; [bola-nEGSQ] = 1.0 × 10^−4^ M.

Self-assembled structures formed by bolaamphiphilic molecules are considered stable.^2^ Using LC-TEM, which enables observation of dynamic behavior in solution,^26^ we elucidated the structure of these assemblies (Fig. 2). The molecular assemblies were found to adopt a monolayer vesicle structure with an internal aqueous domain, consistent in size with that determined by DLS. The membrane thickness was approximately 3.5 nm, corresponding to a single molecular layer (Fig. 3). As shown in Fig. 2d, the size of the monolayer vesicles formed by bola-tetraEGSQ was smaller than the previously reported value (15 nm) for ultra-small monolayer membrane vesicles.^27^ This phenomenon shows that a slight change in the length of nEG groups alters the effective shape of bola-nEGSQ and induces asymmetry in interfacial free energy.^28–30^

While the zeta potential of aggregates formed by amphiphiles with non-ionic ethylene glycol is usually small,^31,32^ it can be seen from Table 1 that their absolute values are large. Specifically, the zeta potentials of the assemblies of bola-monoEGSQ, bola-diEGSQ, and bola-triEGSQ were approximately −30 mV. This suggests that they tend to repel each other, thus maintaining a stable dispersion state.

**Fig. 4:**
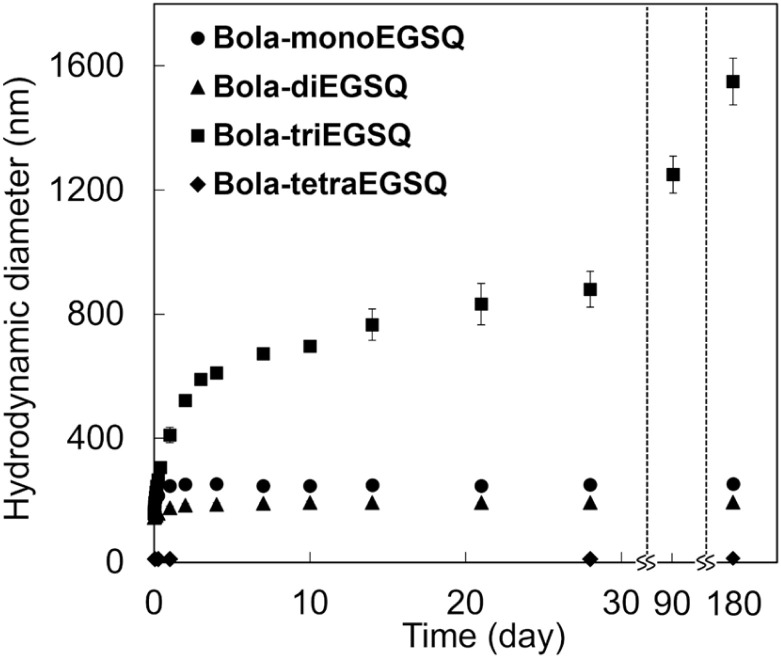
Temporal variation in the diameters of bola-nEGSQ vesicles determined by DLS in water, 25 °C.

Subsequently, the stability of the vesicles over time was investigated. The time-dependent variation in the size of vesicles formed by bola-nEGSQ at a concentration of 1.0 × 10^−4^ M is shown in Fig. 4 and Table S1, SI. Vesicles formed by bola-monoEGSQ and bola-diEGSQ exhibited increases in diameter of approximately 50% and 30%, respectively, over 24 h or 48 h after preparation, and thereafter their diameters remained unchanged after 28 days and even after 180 days. These behaviours may be consistent with an Ostwald ripening mechanism involving molecular dissociation, diffusion, and reassociation; however, in contrast to the bola-triEGSQ system, the bola-mono- and bola-diEGSQ systems are characterised by a rapid cessation of growth within a relatively short timescale. Vesicles with a diameter of 10 nm were formed by bola-tetraEGSQ, and no measurable variation in size was observed even after 180 days, which demonstrates an exceptionally high level of stability. This is likely because the spontaneous curvature induced by the synergy between increased hydrophilicity from the hydration of nEG groups and the packing balance driven by hydrophobic interactions compensates for the curvature elastic energy.^30^ The formation of smaller vesicles upon introduction of tetraethylene glycol units is likely attributable to the increased hydrophilicity of the molecule. The HLB value increases from 7.5 (bola-diEGSQ) to 9.0 (bola-triEGSQ) and 10.1 (bola-tetraEGSQ). The bola-tetraEGSQ derivative is therefore near the boundary between the vesicle-forming region (HLB < 9.4) and the non-aggregating region (HLB > 11.2).^33^ The resulting increase in interfacial curvature is expected to stabilise smaller vesicles while suppressing further growth. While the balance of intermolecular interactions changes continuously with ethylene glycol chain length, the resulting self-assembled structures appear to undergo transitions between distinct regimes. Consequently, the vesicle size and growth behaviour are not monotonic but instead tend to show stepwise changes with respect to the HLB value.

Remarkably, bola-triEGSQ rapidly assembled into vesicles of approximately 160 nm within 5 min of preparation. The vesicles subsequently increased in size to approximately 200 nm after 1 h and reached approximately 410 nm after 24 h, nearly doubling in size. The growth continued gradually, reaching approximately 880 nm after 28 days and further expanding to approximately 1600 nm after 180 days (Table S1, SI). This observation suggests that, under a constant overall bola-triEGSQ concentration, smaller vesicles with higher membrane curvature exhibit relatively greater solubility of the compound. Dissolved molecules likely diffuse and reassemble into larger vesicles, thereby reducing membrane curvature. Such behavior may be attributable to the Gibbs–Thomson effect.^34^

**Fig. 5:**
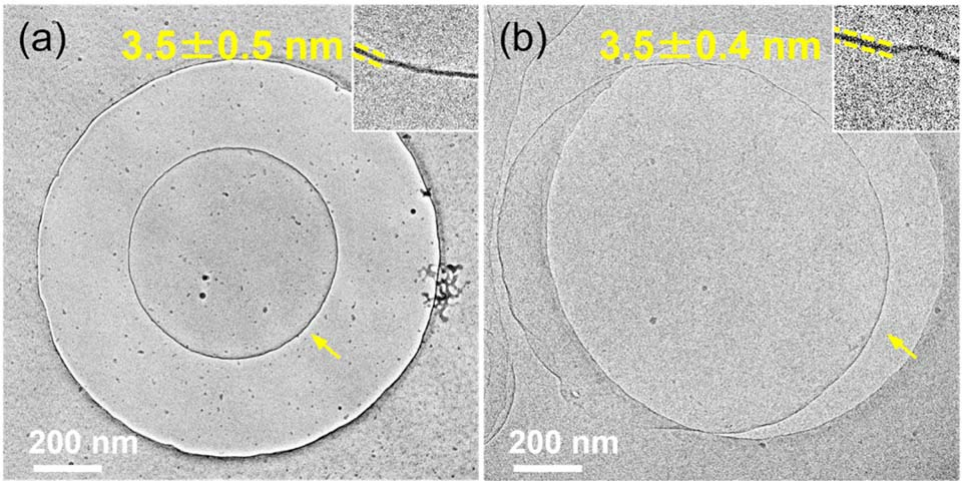
Cryo-TEM images of bola-triEGSQ vesicles at (a) 3 days and (b) 90 days post-mixing.

The zeta potential of all vesicles containing bola-triEGSQ remained essentially unchanged from 1 h to 180 days after preparation (Table S2, SI). This invariance strongly indicates that, despite variations in vesicle size, individual bola-nEGSQ molecules consistently self-assemble into monolayer vesicles, thereby maintaining vesicular composition. Cryo-TEM observations revealed that bola-triEGSQ vesicles retained a well-defined monolayer structure with an approximate thickness of 3.5 nm even after 3 and 90 days (Fig. 5). Similarly, bola-monoEGSQ, bola-diEGSQ, and bola-tetraEGSQ, whose vesicle sizes showed no marked changes over time, were confirmed by LC-TEM on day 180 to maintain the same monolayer vesicle architecture (Fig. S9, SI).

**Fig. 6:**
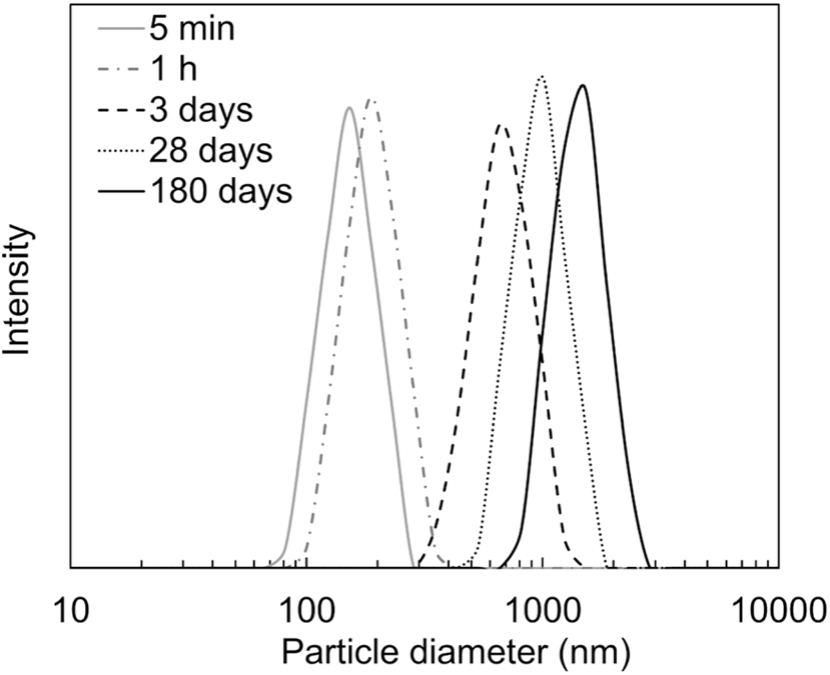
Long-term temporal dynamics of vesicle size distribution (by intensity) formed by bola-triEGSQ at 1.0 × 10^−4^ M.

In composite systems of emulsion nanoparticles, particle size growth has been reported to proceed *via* the Ostwald ripening mechanism.^35–42^ However, no studies to date have demonstrated that vesicles composed of a single species of amphiphilic molecule undergo growth through the same mechanism. Therefore, we examined the growth mechanism of vesicles formed by bola-triEGSQ. The concentration of the substrate in the measurement solution was 1.0 × 10^−4^ M, which is well below the CAC (2.0 × 10^−4^ M). Consequently, the solution remained transparent, indicating that bola-triEGSQ exists either as monomers or small vesicular aggregates in a dissolved state. The evolution of vesicular size distribution and scattering intensity was monitored from 5 min to 180 days post-preparation using DLS (Fig. 6). The mean hydrodynamic diameter gradually increased from approximately 160 nm to 1600 nm over a period of 180 days. These findings indicate a dynamic process wherein individual molecules dissociate from smaller vesicles, diffuse through the medium, and subsequently reassociate to form larger vesicles. It should be noted, however, that the scattering intensity strongly depends on particle size, and larger particles tend to be disproportionately emphasised in DLS measurements. Moreover, for systems spanning the submicron to micrometre range, such as the present one, the scattering deviates from the simple Rayleigh approximation, yet the intensity distribution remains biased towards larger particles. Consequently, when plotted on a logarithmic size scale, distributions that differ in relative width may appear visually similar, even if the actual size distribution evolves over time.

**Fig. 7:**
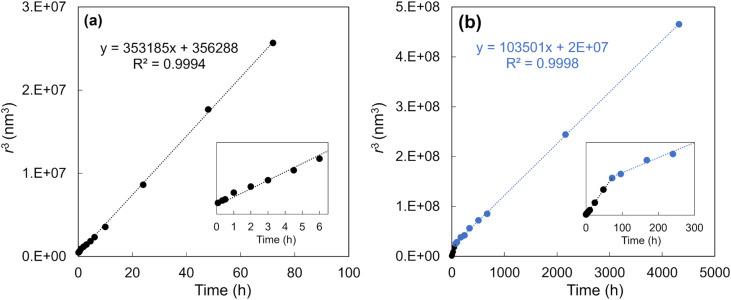
Dependence of the cube of vesicle radius (*r*^3^) formed by bola-triEGSQ on elapsed time at 1.0 × 10^−4^ M; (a) up to 72 h (black circles), (b) after 72 h (blue circles).

According to the Lifshitz–Slyozov–Wagner (LSW) theory, when vesicle growth proceeds *via* the Ostwald ripening mechanism, the growth rate is expected to be diffusion-controlled, and the cube of the mean particle radius should exhibit a linear dependence on time.^43,44^ To verify this prediction, the relationship between the cube of the vesicle radius of bola-triEGSQ and time was plotted based on the data presented in Table S1. Notably, a pronounced linear correlation was observed between these variables (Fig. 7). Such a linear relationship between *r*^3^ and time is consistent with an Ostwald ripening mechanism involving molecular dissociation, diffusion, and reassociation. In contrast, when fusion of smaller vesicles is dominant, non-linear or discontinuous growth behaviour is generally expected. The continuous growth observed over extended periods (up to 180 days) in this study is therefore difficult to account for solely by a fusion mechanism and instead suggests a contribution from growth processes based on molecular transport. However, the involvement of vesicle fusion cannot be completely excluded. Additionally, when the vesicle diameter approached approximately 600 nm (after 72 h), a distinct change in slope occurred. During the initial 72 h period, the slope was *K*_1_ = 3.5 × 10^5^ (Fig. 7a), whereas beyond this point it decreased to *K*_2_ = 1.0 × 10^5^ (Fig. 7b), representing a reduction to roughly one-third of its original value.

Thus, the vesicle growth process exhibited a biphasic transition in rate, progressively shifting toward a slower regime. On this basis, we propose an explicit model in which molecular transport remains diffusion-controlled throughout the process, while the rate constant is not invariant but varies in response to the magnitude of the driving force. During the initial stage, the curvature distribution across the vesicular surface is markedly heterogeneous, leading to a substantial increase in Laplace pressure and solubility differences. These pressure differentials promote molecular migration *via* the Gibbs–Thomson mechanism, thereby amplifying the diffusion flux; consequently, the apparent rate constant *K*_1_ attains a relatively high magnitude. In contrast, the later stage is characterized by interfacial homogenization through molecular rearrangement, which diminishes curvature disparities and solubility differences, thereby attenuating the driving force. Although the process remains diffusion-controlled, the rate constant satisfies the relation *K*_2_ < *K*_1_.^45,46^

This biphasic model offers a novel interpretative framework that preserves the conceptual foundation of LSW theory while quantitatively explaining the experimentally observed transition from rapid to gradual growth. Nevertheless, the observed inflection in vesicular growth kinetics near a diameter of approximately 600 nm is likely attributable to curvature-dependent effects on the membrane, a hypothesis that is currently under investigation.^47^

**Fig. 8:**
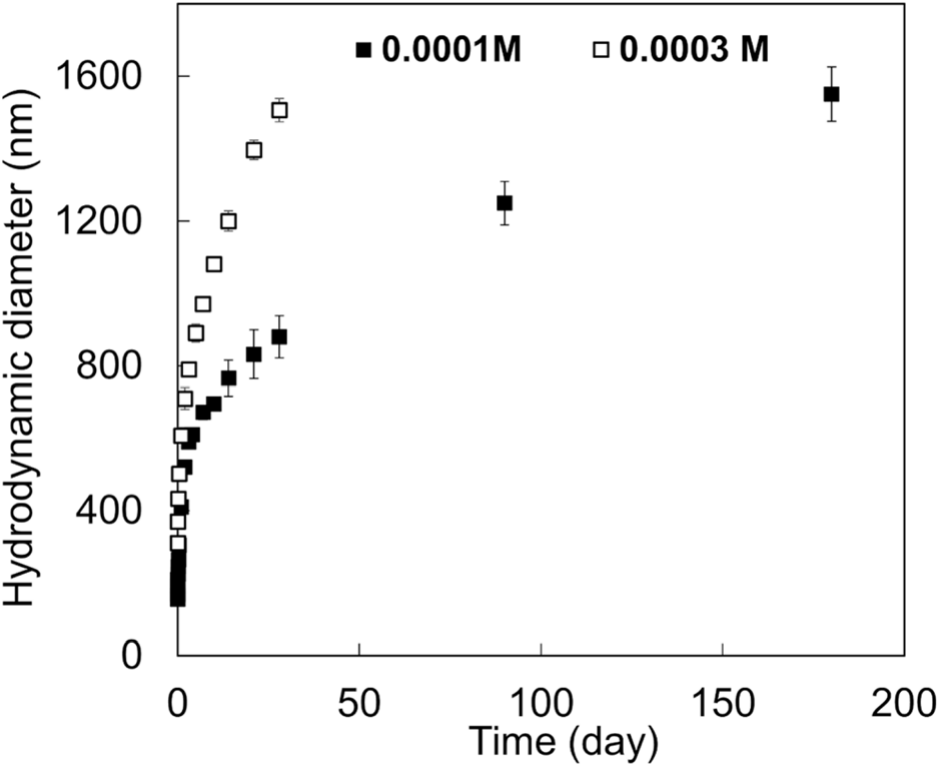
Temporal variation in the diameter of bola-triEGSQ vesicles determined by DLS in water, 25 °C.

**Fig. 9:**
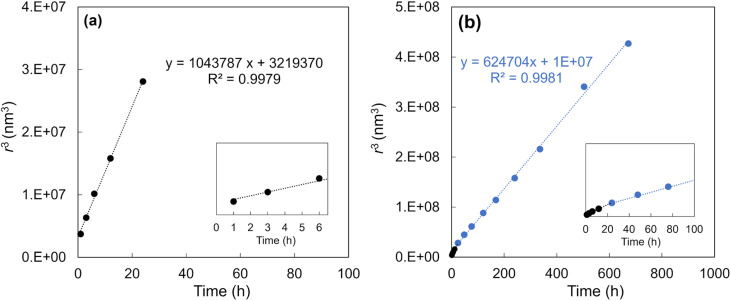
Dependence of the cube of vesicle radius (*r*^3^) formed by bola-triEGSQ on elapsed time at 3.0 × 10^−4^ M; (a) up to 24 h (black circles); (b) after 24 h (blue circles).

Given that the Ostwald ripening mechanism has been postulated to operate under diffusion-limited molecular processes, the bola-triEGSQ concentration was increased to three times the standard level (3.0 × 10^−4^ M), and the temporal evolution of vesicular diameter was systematically monitored (Fig. 8 and Table S3, SI). Under these conditions, the growth rate exhibited a pronounced acceleration: whereas a diameter of 1500 nm typically requires approximately 180 days to achieve at the concentration of 1.0 × 10^−4^ M, it was attained within only 28 days. Cryo-TEM observations revealed no discernible concentration-dependent variation in vesicular morphology; instead, a monolayer vesicle architecture was consistently observed, with a membrane thickness of approximately 3.5 nm (Fig. S10, SI). To elucidate the growth kinetics, the time-dependent variation in the cube of the vesicle radius was quantified over the initial 28 days (Fig. 9). As in the low-concentration regime, a robust linear correlation was evident, although the slope (*K*) underwent a distinct transition when the vesicle diameter approached approximately 610 nm. During the first 24 h, the vesicle growth rate constant *K* increased by approximately threefold, and the vesicle diameter reached approximately 610 nm. Thereafter, the diameter exhibited gradual expansion to 1500 nm over 28 days, a behavior that appears to align with predictions derived from LSW theory.

## Conclusions

From these results, we elucidate that the bolaamphiphiles, functionalized with ethylene glycol groups at both terminal double bonds of squalene, self-assemble into vesicular structures whose curvature is governed by the length of the ethylene glycol groups. LC-TEM and Cryo-TEM observations unequivocally visualized that these vesicular structures consistently adopt monolayer vesicles, irrespective of monomeric configuration or aggregate size. Remarkably, bola-triEGSQ exhibits Ostwald ripening growth dynamics, requiring approximately six months to mature into vesicles with a diameter of 1600 nm, a process that can be interpreted as diffusion-limited. Intriguingly, within this system, we identified for the first time a two-step mechanism characterized by two distinct diffusion coefficients, each dominated by different driving forces. This finding provides divergent insight into the kinetic complexity underlying amphiphilic self-assembly. Current efforts focus on achieving precise regulation of size and growth kinetics under external stimuli.

## Author contributions

Tran Ngoc Linh: conceptualization, methodology, investigation, validation, writing – original draft, writing – review & editing. Rintarou Ootani: investigation, writing – review & editing. Hirohmi Watanabe: writing – review & editing. Takashi Arimura: conceptualization, methodology, writing – original draft, writing – review & editing, funding acquisition, supervision, visualization. Hiroko Isoda: investigation, writing – review & editing. Masato Kawasaki: investigation, writing – review & editing.

## Conflicts of interest

There are no conflicts to declare.

## Supplementary Material

RA-OLF-D6RA03150A-s001

## Data Availability

The data supporting this article have been included as part of the supplementary information (SI). The authors have cited additional references within the SI.^48^ Supplementary information: the experimental details, synthetic procedures, NMR data, additional LC-TEM and Cryo-TEM images. See DOI: https://doi.org/10.1039/d6ra03150a.
