# Supplementary material for: Divergent self-assembly dynamics of squalene-derived bolaamphiphiles enabled by ethylene glycol-length-switchable Ostwald ripening
Source: RSC Adv. 2026 Jul 3;16(34):31856–63. doi: 10.1039/d6ra03150a (PMC13329817; doi:10.1039/d6ra03150a)
Supplement: RA-016-D6RA03150A-s001 [file RA-016-D6RA03150A-s001.pdf]

Supplementary Information (SI) for RSC Advances.
This journal is © The Royal Society of Chemistry 2026

Supplementary Information (SI) for

Divergent self-assembly dynamics of squalene-derived bolaamphiphiles enabled by ethylene glycol–length–switchable Ostwald ripening

Tran Ngoc Linh,[a] Rintarou Ootani,[b] Hirohmi Watanabe,[a] Takashi Arimura,*[b] Hiroko Isoda,[b,c] and Masato Kawasaki[d]

[a] Research Institute for Sustainable Chemistry, National Institute of Advanced Industrial Science and Technology (AIST), 3-11-32, Kagamiyama, Higashihiroshima, Hiroshima 739-0046, Japan.
[b] Open Innovation Laboratory for Food and Medicinal Resource Engineering (FoodMed-OIL), National Institute of Advanced Industrial Science and Technology (AIST), Laboratory of Advanced Research D, University of Tsukuba, Tsukuba, Ibaraki 305-0006, Japan.
[c] Institute of Life and Environmental Sciences, University of Tsukuba, Tsukuba, Ibaraki 305-8572, Japan.
[d] Institute of Materials Structure Science, Inter-University Research Institute Corporation High Energy Accelerator Research Organization (KEK), 1-1 Oho, Tsukuba, Ibaraki 305-0801, Japan.

* Corresponding author:
Takashi Arimura
FoodMed-OIL, AIST, Laboratory of Advanced Research D, University of Tsukuba, Tsukuba, Ibaraki 305-0006, Japan.
Tel: (+81) 50-3521-3255
E-mail: takashi-arimura@aist.go.jp

1 Experiments and Methods

General information. New compounds were characterized by NMR spectroscopy (^{1}H NMR, ^{13}C NMR), high-resolution mass spectra (HRMS) and Fourier transform infrared spectroscopy (FTIR). NMR spectra were recorded on a JEOL 400YH spectrometer operating 400 MHz at 25 ºC in Fourier transform mode. ^{1}H NMR spectra were reported in *delta* units, parts per million (ppm), and were calibrated using 1,4-bis(trimethylsilyl)benzene-d_4 (1,4-BTMSB-d_4) as an internal standard (0 ppm) in chloroform-d_1 ($CDCl_3$). ^{13}C NMR spectrum was reported in ppm with reference to 1,4-BTMSB-d_4 (0 ppm) and was recorded with ^{1}H decoupling. HRMS analysis was performed using electron ionization (EI) on a JEOL JMS-700 mass spectrometer. IR spectrum was recorded in attenuated total refraction (ATR) mode on a JASCO FT-IR 4100 spectrometer.

Purities (*P*, %) of all compounds were determined by quantitative NMR (qNMR) according to following equation.[S1]

$$P = \frac{Int_{\mathrm{t}}}{Int_{\mathrm{IC}}} \times \frac{n_{\mathrm{IC}}}{n_{\mathrm{t}}} \times \frac{M_{\mathrm{t}}}{M_{\mathrm{IC}}} \times \frac{m_{\mathrm{IC}}}{m_{\mathrm{t}}} \times P_{\mathrm{IC}}$$

Where:

Int_{IC} : area (integral) of the internal calibrant (IC) resonance signal

Int_{t} : area (integral) of the target analyte (t) resonance signal

n_{IC} : number of protons of the internal calibrant that give rise to Int_{IC}

n_{t} : number of protons of the target analyte that give rise to Int_{t}

M_{IC} : molecular weight of the internal calibrant (IC)

M_{t} : molecular weight of the target analyte (t)

m_{IC} : weight (mass) of the internal calibrant (IC)

m_{t} : weight (mass) of the target analyte (t)

P_{IC} : purity of the internal calibrant, as percentage value

Materials. Solvents were reagent grade and used as received unless otherwise noted. All column chromatography was performed using Wako silica gel C-300 as the stationary phase.

Critical aggregation concentration (CAC) measurements

Bolaamphiphiles dissolved in ethanol were injected into a solution containing pyrene (concentration of 5.0 × 10^{-7} M). The fluorescence measurements were conducted on a RF-5300PC spectrofluorometer (Shimadzu, Japan) at 25 ºC, using an excitation wavelength of 335 nm. The variation of intensity ratio of band III (at 384 nm) to band I (at 373 nm) of pyrene fluorescence emission spectra, was examined with increasing concentration of the bolaamphiphiles. All the experiments were performed in triplicate.

Particle size and zeta potential measurements

Particle size measurements were conducted using dynamic light scattering (DLS) on ELSZneo equipment (Otsuka Electronics, Japan) at 25 °C. The hydrodynamic diameter (*D*) and polydispersity index (PDI) values are expressed as mean ± SD of at least three independent experiments. Zeta potential measurements were conducted using laser doppler electrophoresis on the same ELSZneo equipment at 25 °C. The zeta potential (ζ) values were calculated using the Smoluchowski equation and the values are expressed as mean ± SD of at least three independent experiments.

Liquid cell transmission electron microscopy (LC-TEM)

A small volume of the liquid sample was loaded into a liquid cell (K-kit, Bio MA-TEK) by capillary action, followed by being sealed with water-resistant glue. TEM images were acquired at an accelerating voltage of 200 kV using a JEM-ARM200F NEOARMex (JEOL, Japan). TEM images were analyzed using ImageJ software (National Institute of Health, USA).

Cryogenic transmission electron microscopy (Cryo-TEM)

A droplet (3 μL) of the sample was pipetted onto a carbon-coated copper grid loaded into an FEI Vitrobot apparatus. The grid was blotted for approximately 15 s at 18 °C and 100% humidity, then rapidly plunged into liquid ethane (~90 K) cooled by liquid nitrogen. The sample was transferred to a cryoholder mounted on a cryotransfer stage immersed in liquid nitrogen. TEM images were acquired at an accelerating voltage of 200 kV using a Talos Arctica (Thermo Fisher Scientific, USA) at the Cryo-EM facility of the High Energy Accelerator Research Organization (KEK), Tsukuba, Japan. TEM images were analyzed using ImageJ software.

Hydrophilic-lipophilic balance (HLB) of bolaamphiphiles

HLB (= 20 × $MW_{\_hydrophilic}$ / $MW_{\_total}$) values, calculated by the Griffin method,[S2] are 5.5 for **bola-monoEGSQ**, 7.5 for **bola-diEGSQ**, 9.0 for **bola-triEGSQ**, and 10.1 for **bola-tetraEGSQ**.

2 Synthetic procedures

2,23-bis(2-hydroxyethoxy)-3,22-dihydroxysqualene (bola-monoEGSQ)

2,3;22,23-Diepoxysqualene was prepared according to the reported method.[S3,S4]

2,3;22,23-Diepoxysqualene (100 mg, 0.23 mmol) and mono-ethylene glycol (8.57 g, 138 mol) were dissolved in 2-propanol (5 mL). The mixture was heated at 80 ºC in 6 h with stirring and cooled to room temperature. Then, water (100 mL) and ethyl acetate (150 mL) were added, and the organic layer was washed with saturated brine and water. The solvent was evaporated and purification by column chromatography on silica gel eluting with hexane/ethyl acetate gave colorless oil **bola-monoEGSQ** (91 mg; 70% of isolated yield; 96% of purity by qNMR with 1,4-BTMSB-d_4 as an internal standard).

^{1}H NMR (400 MHz, $CDCl_3$): δ 5.03–4.76 (m, 4H), 3.47–3.43 (m, 4H), 3.41–3.11 (m, 8H), 2.05–1.98 (m, 2H), 1.89–1.61 (m, 16H), 1.34 (br s, 12H), 1.25–1.13 (m, 4H), 0.88 (s, 6H), 0.87 (s, 6H) ppm.

^{13}C NMR (101 MHz, $CDCl_3$): δ 136.2, 136.0, 125.9, 125.5, 79.0, 78.4, 77.2, 63.6, 63.4, 40.9, 38.0, 30.9, 29.4, 27.8, 22.8, 21.0, 17.2 ppm.

HRMS (EI): m/z $[M]^+$ calculated for $C_{34}H_{62}O_6$ 566.4546; found 566.4531.

FTIR (neat): 3381, 2969, 2931, 2870, 1735, 1455, 1381, 1365, 1216, 1146, 1076, 968, 939, 889 cm^{-1}.

2,23-bis(2-(2-hydroxyethoxy)ethoxy)-3,22-dihydroxysqualene (bola-diEGSQ)

2,3;22,23-Diepoxysqualene (100 mg, 0.23 mmol) and di-ethylene glycol (14.6 g, 138 mol) were dissolved in 2-propanol (5 mL). The mixture was heated at 80 ºC in 6 h with stirring and cooled to room temperature. Then, water (100 mL) and ethyl acetate (150 mL) were added, and the organic layer was washed with saturated brine and water. The solvent was evaporated and purification by column chromatography on silica gel eluting with hexane/ethyl acetate gave colorless oil **bola-diEGSQ** (101 mg; 67% of isolated yield; 97% of purity by qNMR with 1,4-BTMSB-d_4 as an internal standard).

^{1}H NMR (400 MHz, $CDCl_3$): δ 4.98–4.77 (m, 4H), 3.49–3.44 (m, 4H), 3.44–3.30 (m,12H), 3.29–3.23 (m, 4H), 2.07–2.00 (m, 2H), 1.91–1.65 (m, 16H), 1.34 (br s, 12H), 1.20–1.12 (m, 4H), 0.89 (s, 6H), 0.86 (s, 6H) ppm.

^{13}C NMR (101 MHz, $CDCl_3$): δ 136.3, 136.1, 125.8, 125.5, 79.3, 78.4, 75.8, 73.9, 72.0, 63.0, 61.8, 40.9, 38.2, 31.0, 29.5, 27.9, 22.9, 21.5, 17.3 ppm.

HRMS (EI): m/z $[M]^+$ calculated for $C_{38}H_{70}O_8$ 654.5071; found 654.5090.

FTIR (neat): 3395, 2969, 2932, 2872, 1718, 1455, 1381, 1365, 1221, 1125, 1066, 972, 924, 884 cm^{-1}.

2,23-bis(2-(2-(2-hydroxyethoxy)ethoxy)ethoxy)-3,22-dihydroxysqualene (bola-triEGSQ)

2,3;22,23-Diepoxysqualene (100 mg, 0.23 mmol) and tri-ethylene glycol (20.7 g, 138 mol) were dissolved in 2-propanol (5 mL). The mixture was heated at 80 ºC in 6 h with stirring and cooled to room temperature. Then, water (100 mL) and ethyl acetate (200 mL) were added, and the organic layer was washed with saturated brine and water. The solvent was evaporated and purification by column chromatography on silica gel eluting with hexane/ethyl acetate gave colorless oil **bola-triEGSQ** (116 mg; 68% of isolated yield; 95% of purity by qNMR with 1,4-BTMSB-d_4 as an internal standard).

^{1}H NMR (400 MHz, $CDCl_3$): δ 5.03–4.74 (m, 4H), 3.47–3.43 (m, 4H), 3.43–3.39 (m, 6H), 3.39–3.31 (m, 16H), 3.25–3.20 (m, 2H), 2.09–2.00 (m, 2H), 1.94–1.55 (m, 16H), 1.34 (br s, 12H), 1.18–1.08 (m, 4H), 0.86 (s, 6H), 0.85 (s, 6H) ppm.

^{13}C NMR (101 MHz, $CDCl_3$): δ 136.4, 136.2, 125.6, 125.4, 79.1, 78.4, 74.7, 74.5, 71.81, 71.76, 71.1, 62.9, 61.5, 41.0, 38.2, 31.1, 29.5, 28.0, 23.1, 21.8, 17.3 ppm.

HRMS (EI): m/z $[M]^+$ calculated for $C_{42}H_{78}O_{10}$ 742.5595; found 742.5601.

FTIR (neat): 3433, 2969, 2922, 2870, 1717, 1455, 1381, 1364, 1230, 1121, 1077, 971, 933, 885 cm^{-1}.

2,23-bis(2-(2-(2-(2-hydroxyethoxy)ethoxy)ethoxy)ethoxy)-3,22-dihydroxysqualene (bola-tetraEGSQ)

2,3;22,23-Diepoxysqualene (100 mg, 0.23 mmol) and tetra-ethylene glycol (26.8 g, 138 mol) were dissolved in 2-propanol (5 mL). The mixture was heated at 80 ºC in 6 h with stirring and cooled to room temperature. Then, water (100 mL) and ethyl acetate (200 mL) were added, and the organic layer was washed with saturated brine and water. The solvent was evaporated and purification by column chromatography on silica gel eluting with hexane/ethyl acetate gave colorless oil **bola-tetraEGSQ** (130 mg; 68% of isolated yield; 97% of purity by qNMR with 1,4-BTMSB-d_4 as an internal standard).

^{1}H NMR (400 MHz, $CDCl_3$): δ 4.97–4.78 (m, 4H), 3.47–3.44 (m, 4H), 3.41–3.38 (m, 18H), 3.36–3.31 (m, 10H), 3.27–3.22 (m, 4H), 2.09–2.00 (m, 2H), 1.95–1.56 (m, 16H), 1.34 (br s, 12H), 1.19–1.12 (m, 4H), 0.87 (s, 6H), 0.85 (s, 6H) ppm.

^{13}C NMR (101 MHz, $CDCl_3$): δ 136.4, 136.2, 125.6, 125.4, 79.1, 78.4, 76.1, 73.9, 72.1, 71.81, 71.76, 71.6, 71.5, 62.8, 61.7, 41.0, 38.2, 31.1, 29.5, 28.0, 23.0, 21.2, 17.3 ppm.

HRMS (ESI): m/z $[M + Na]^+$ calculated for $C_{46}H_{86}O_{12}Na$ 853.6017; found 853.6008.

FTIR (neat): 3446, 2969, 2911, 2869, 1735, 1455, 1380, 1364, 1217, 1125, 1078, 973, 940, 885 cm^{-1}.

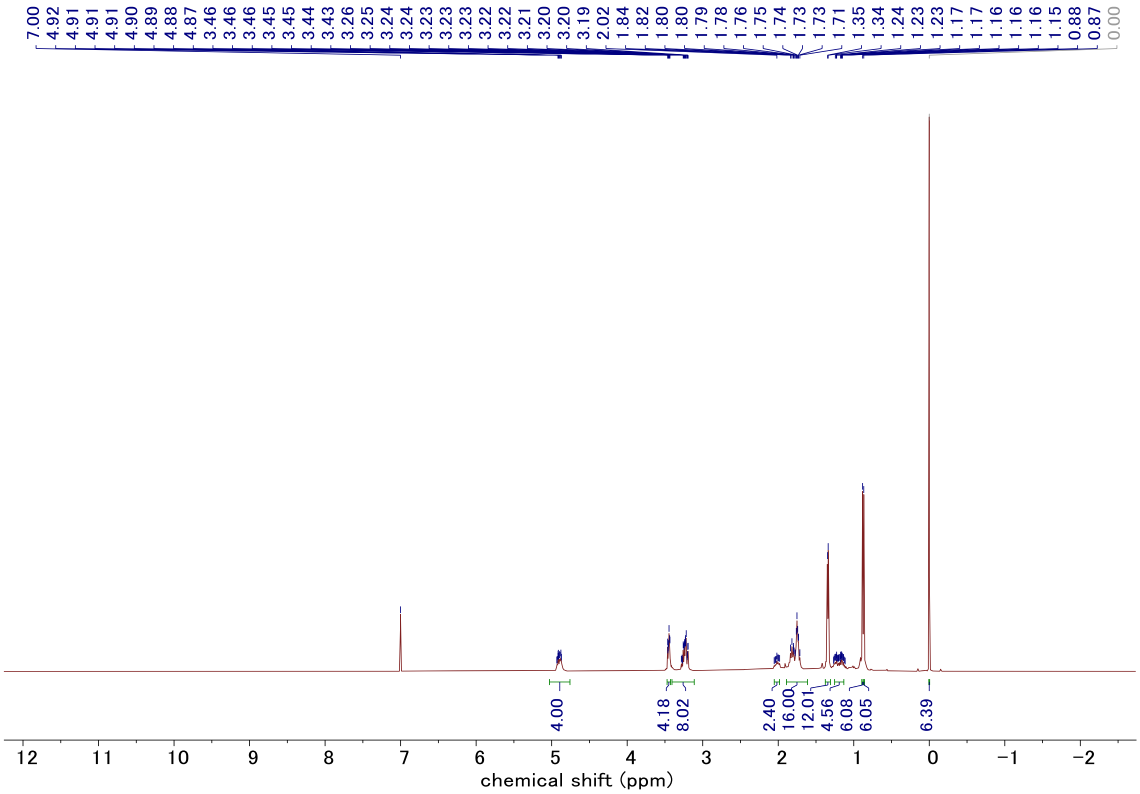

Fig. S1 ^{1}H NMR spectrum of **bola-monoEGSQ** (400 MHz, 25 °C, $CDCl_3$).

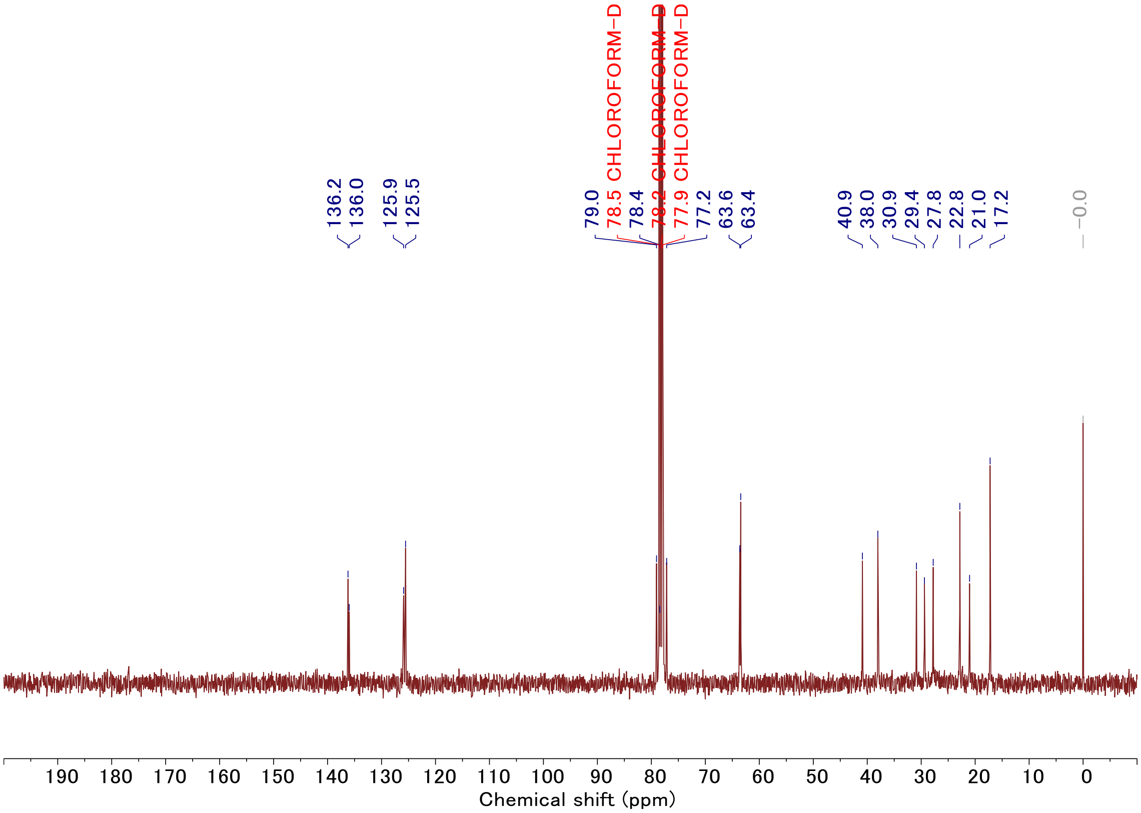

Fig. S2 ^{13}C NMR spectrum of **bola-monoEGSQ** (101 MHz, 25 °C, $CDCl_3$).

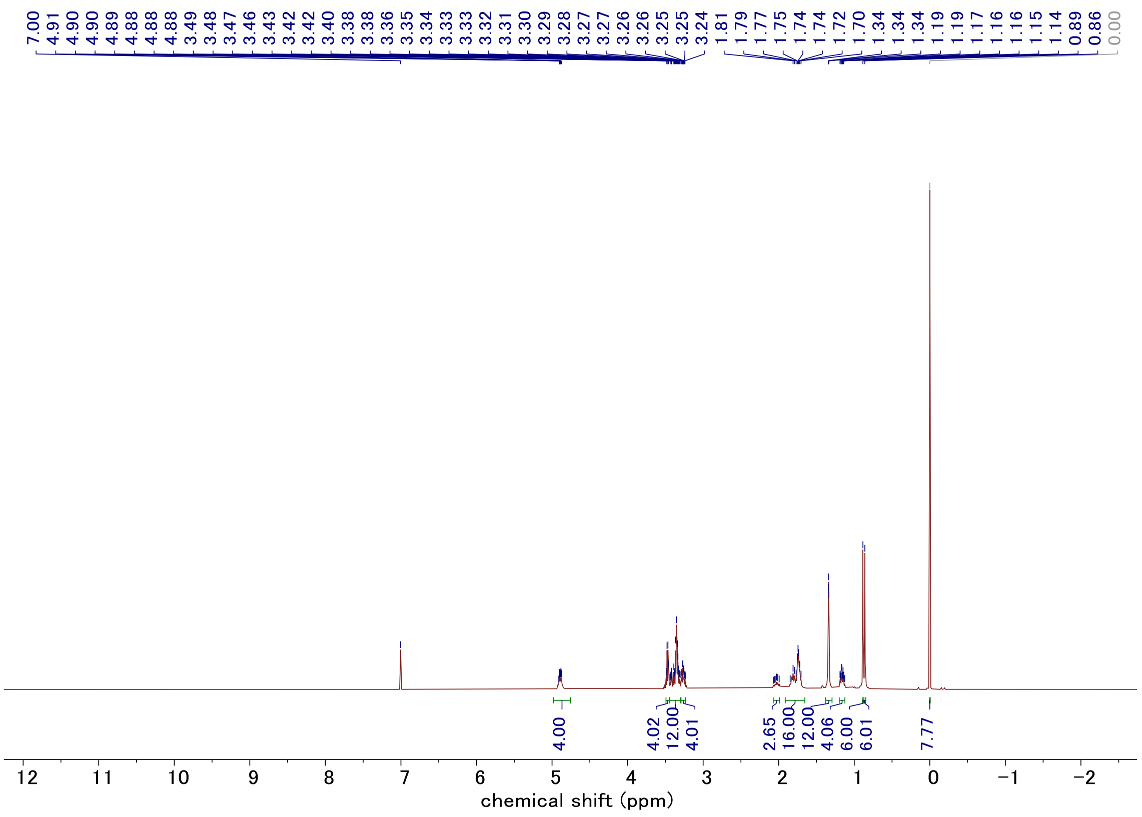

Fig. S3 ^{1}H NMR spectrum of **bola-diEGSQ** (400 MHz, 25 °C, $CDCl_3$).

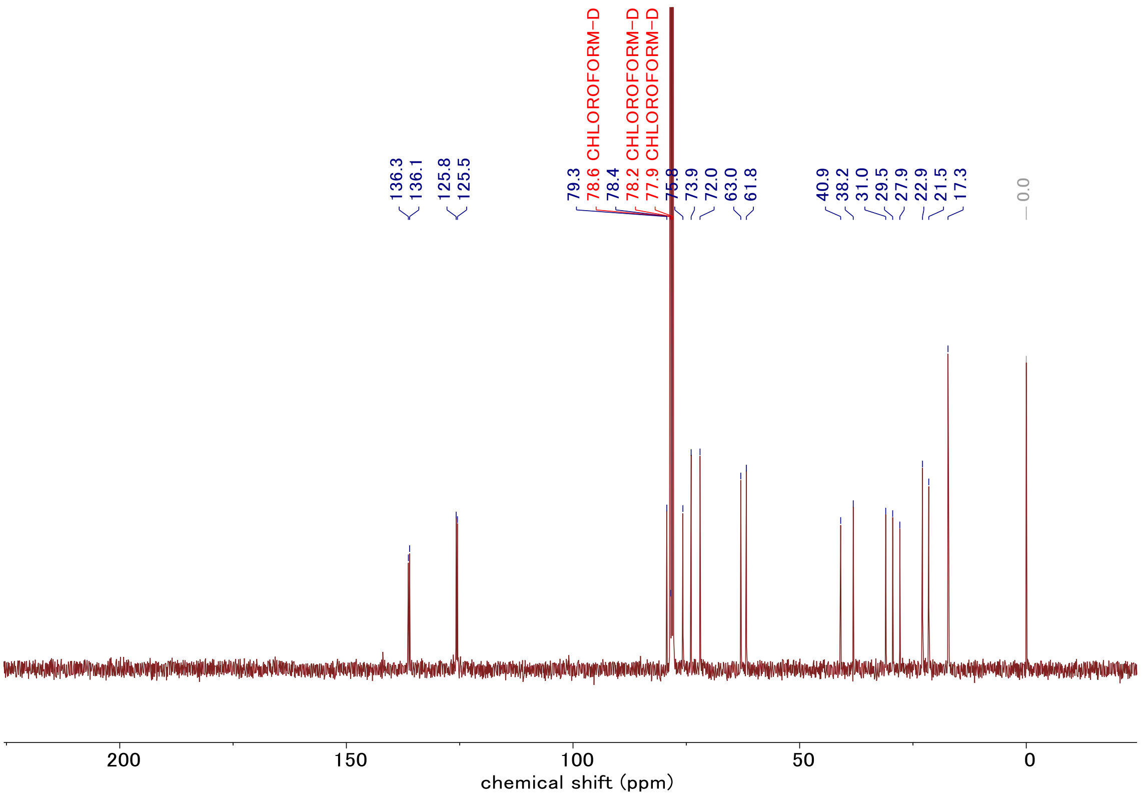

Fig. S4 ^{13}C NMR spectrum of **bola-diEGSQ** (101 MHz, 25 °C, $CDCl_3$).

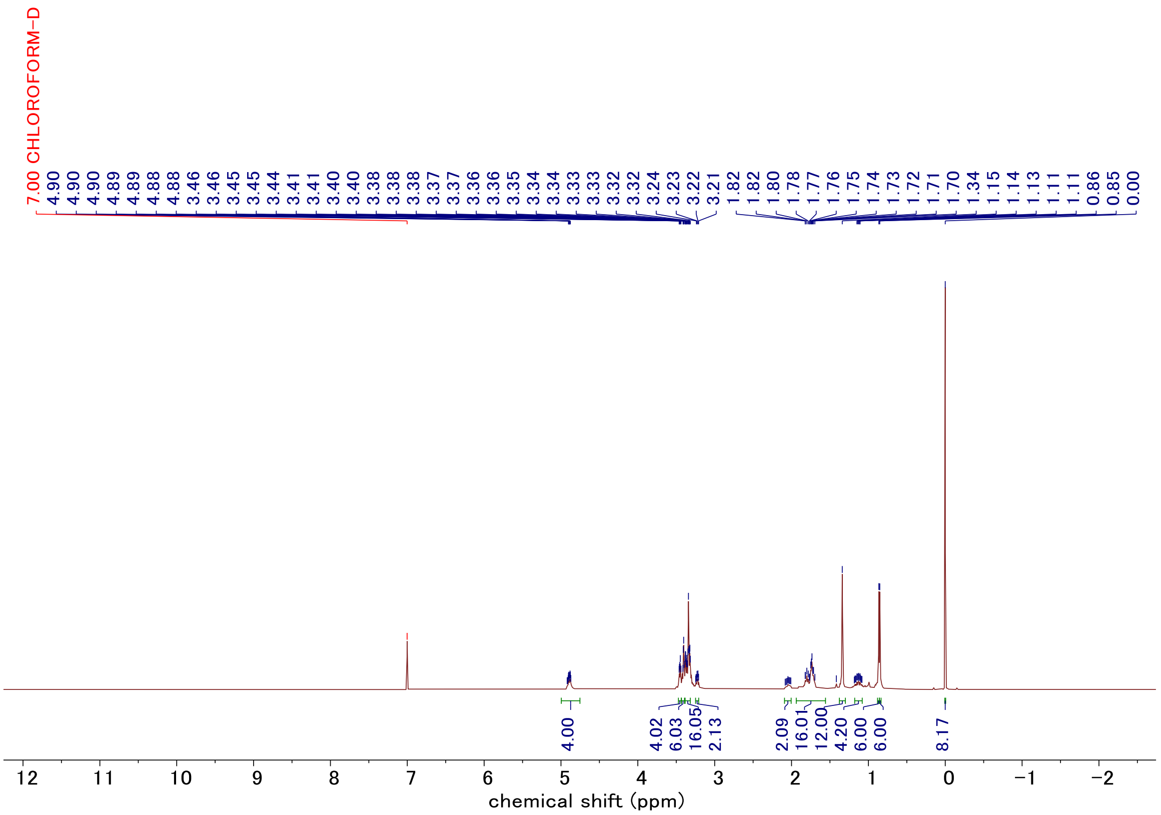

Fig. S5 ^{1}H NMR spectrum of **bola-triEGSQ** (400 MHz, 25 °C, $CDCl_3$).

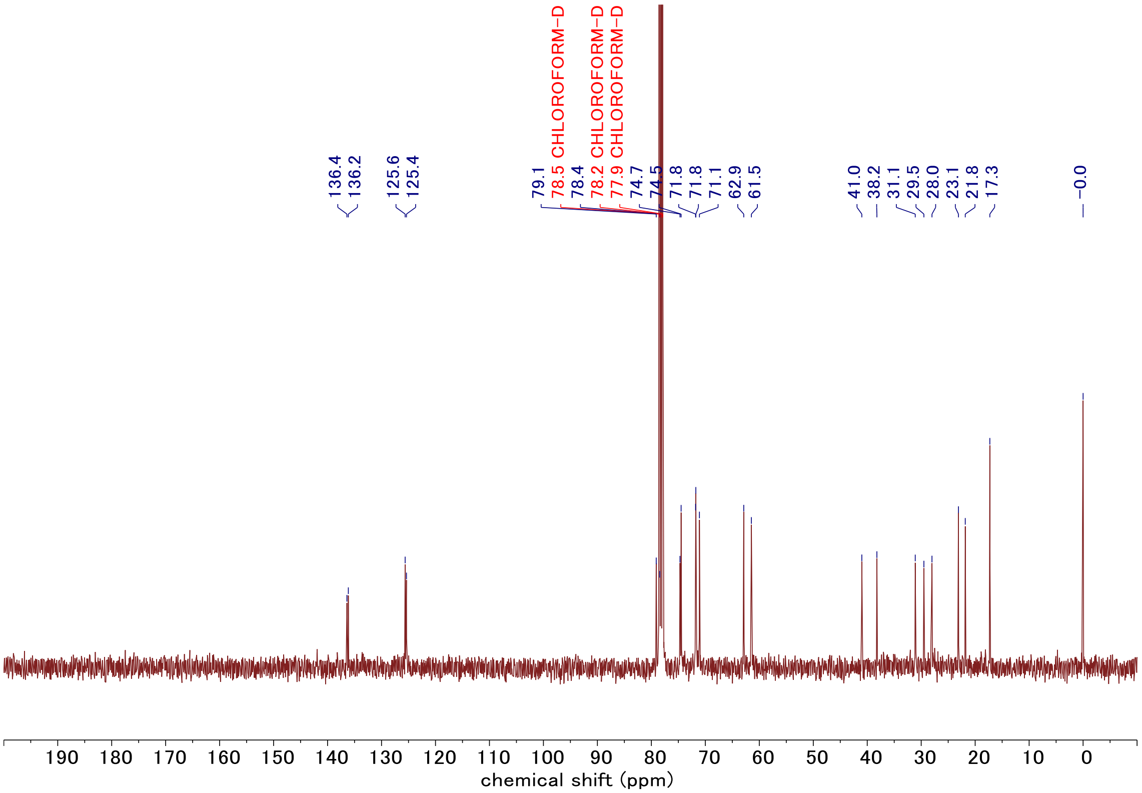

Fig. S6 ^{13}C NMR spectrum of **bola-triEGSQ** (101 MHz, 25 °C, $CDCl_3$).

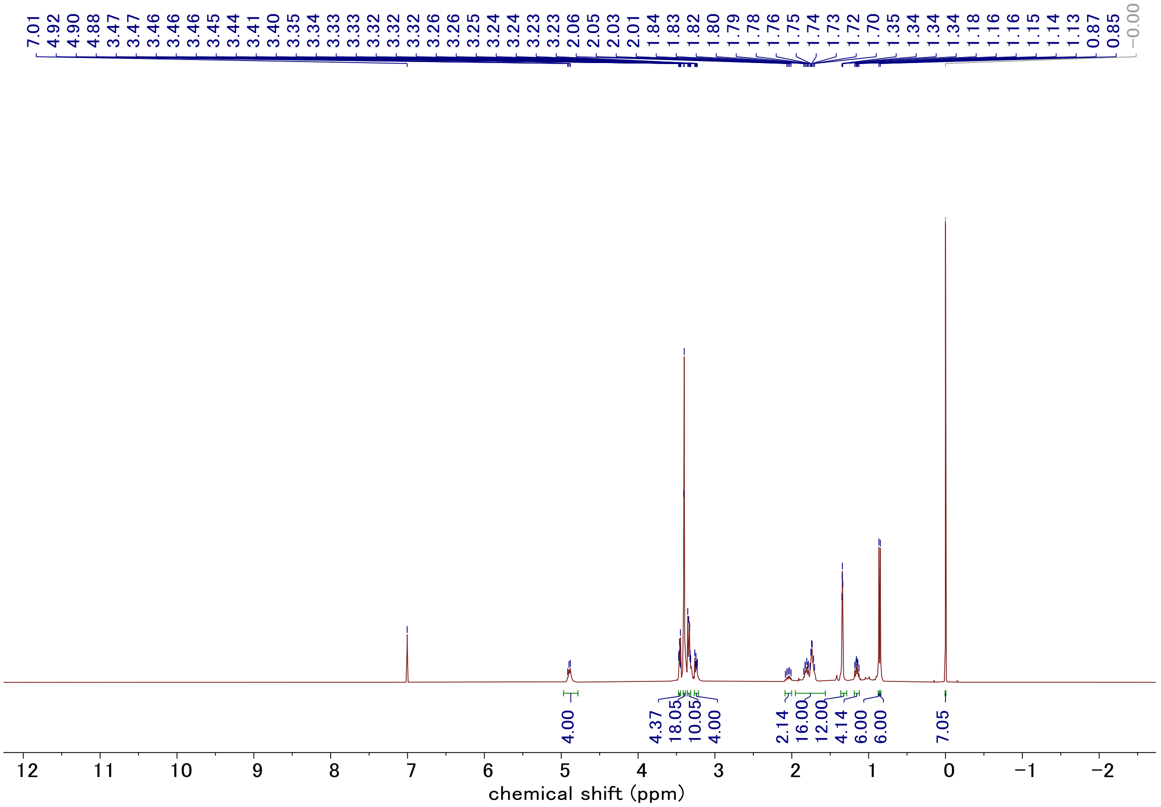

Fig. S7 ^{1}H NMR spectrum of **bola-tetraEGSQ** (400 MHz, 25 °C, $CDCl_3$).

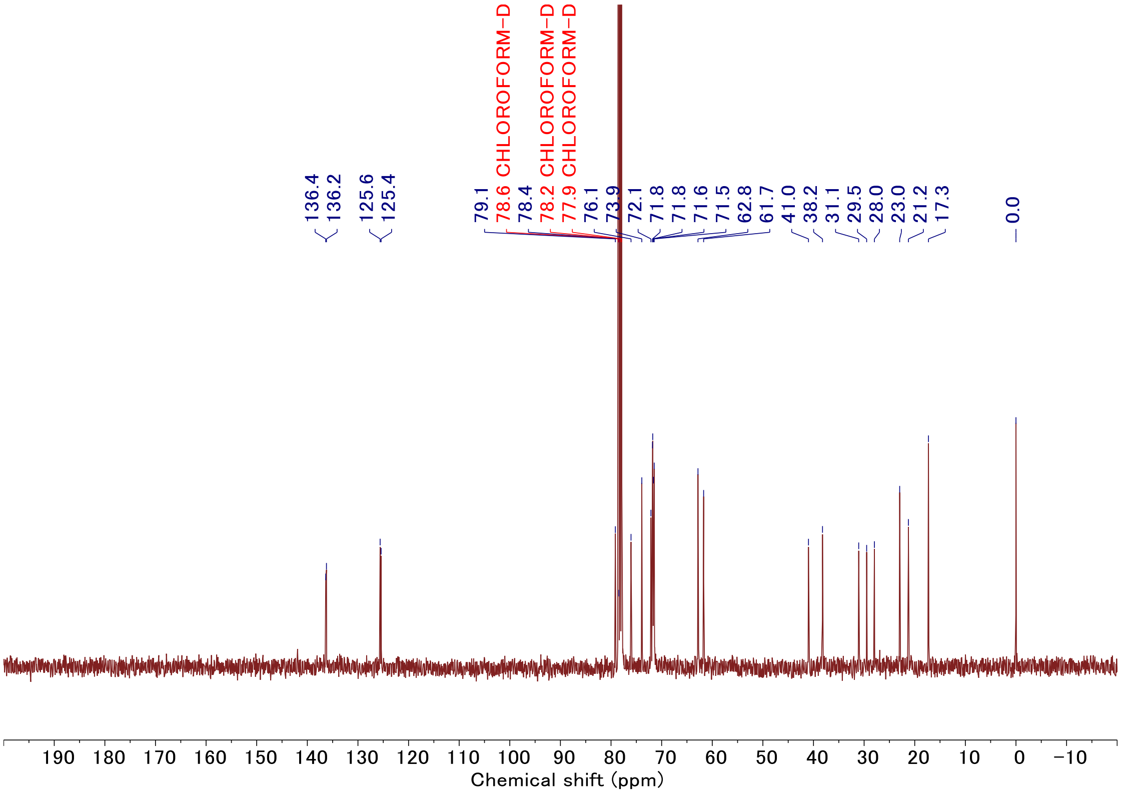

Fig. S8 ^{13}C NMR spectrum of **bola-tetraEGSQ** (101 MHz, 25 °C, $CDCl_3$).

Table S1 Vesicle size variation over time for four bolaamphiphiles (**bola-nEGSQ**) determined by DLS in water, 25 ºC; [**bola-nEGSQ**] = 1.0 × 10^{-4} M

Time (h)	Hydrodynamic diameter (*D*, nm)			
	**bola-monoEGSQ**	**bola-diEGSQ**	**bola-triEGSQ**	**bola-tetraEGSQ**
0.08	166 ± 3	144 ± 1	156 ± 6	
0.33	179 ± 2	145 ± 2	166 ± 6	
0.5	185 ± 3	146 ± 2	171 ± 7	
1	194 ± 2	147 ± 4	193 ± 8	10 ± 2
2			210 ± 3	
3	207 ± 1	151 ± 2	225 ± 5	
4.5			245 ± 8	
6	215 ± 5	157 ± 6	265 ± 10	10 ± 1
10			305 ± 11	
24 (1 day)	247 ± 5	176 ± 6	410 ± 25	11 ± 1
48 (2 days)	251 ± 5	184 ± 6	521 ± 15	
72 (3 days)			590 ± 10	
96 (4 days)	253 ± 6	187 ± 5	610 ± 18	
168 (7 days)	246 ± 2	190 ± 3	672 ± 20	
240 (10 days)	247 ± 5	192 ± 5	696 ± 17	
336 (14 days)	249 ± 3	192 ± 3	766 ± 50	
504 (21 days)	247 ± 9	193 ± 1	832 ± 67	
672 (28 days)	250 ± 10	193 ± 0	880 ± 58	11 ± 2
2160 (90 days)			1250 ± 60	
4320 (180 days)	252 ± 8	192 ± 6	1550 ± 75	11 ± 2

Table S2 Zeta potential variation over time for vesicles of four bolaamphiphiles (**bola-nEGSQ**) in water, 25 ºC; [**bola-nEGSQ**] = 1.0 × 10^{-4} M

Time (h)	Zeta potential (ζ, mV)			
	**bola-monoEGSQ**	**bola-diEGSQ**	**bola-triEGSQ**	**bola-tetraEGSQ**
1	−31 ± 4	−33 ± 3	−27 ± 2	−15 ± 1
24 (1 day)			−28 ± 1	
168 (7 days)	−33 ± 1	−33 ± 2	−30 ± 3	
672 (28 days)			−25 ± 1	
4320 (180 days)	−31 ± 3	−34 ± 2	−29 ± 1	−16 ± 2

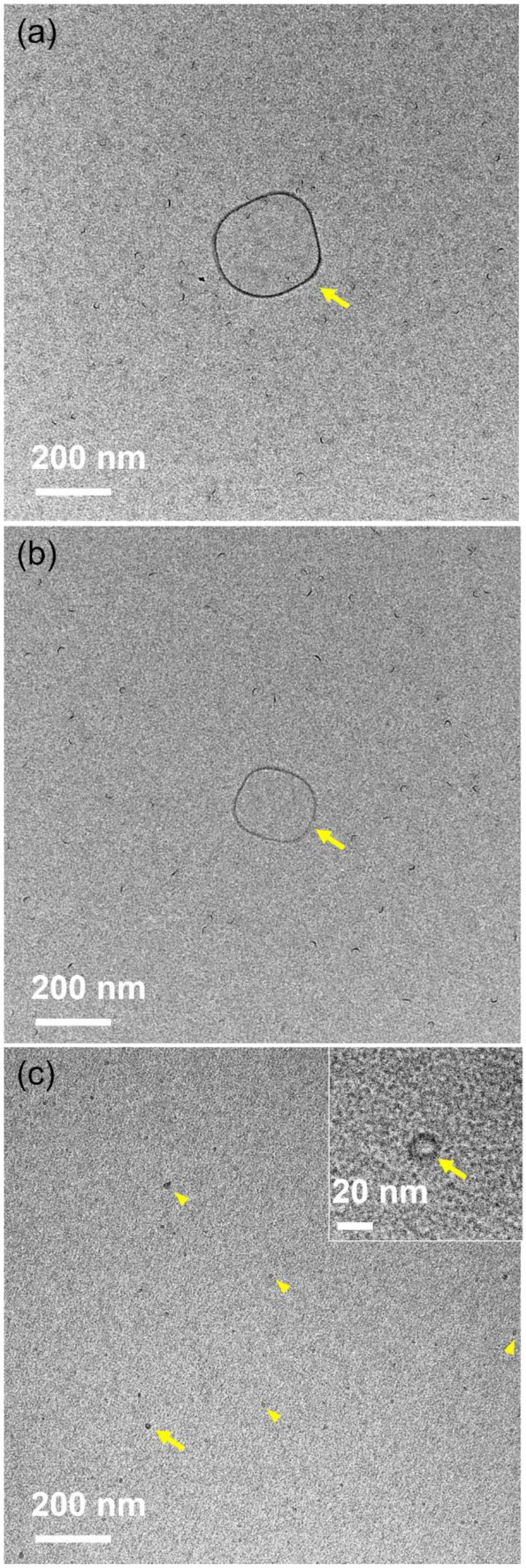

Fig. S9 LC-TEM images of (a) **bola-monoEGSQ**, (b) **bola-diEGSQ**, (c) **bola-tetraEGSQ** vesicles in water, 25 ºC at 180 days post-mixing; [**bola-nEGSQ**] = 1.0 × 10^{-4} M.

Table S3 Vesicle size variation over time for **bola-triEGSQ** determined by DLS in water, 25 ºC; [**bola-triEGSQ**] = 3.0 × 10^{-4} M

Time (h)	Hydrodynamic diameter (D, nm)
	**bola-triEGSQ**
1	310 ± 13
3	370 ±15
6	433 ± 10
12	502 ± 7
24 (1 day)	608 ± 22
48 (2 days)	710 ± 30
76 (3.2 days)	789 ± 10
120 (5 days)	890 ± 25
168 (7 days)	970 ± 11
240 (10 days)	1081 ± 16
336 (14 days)	1200 ± 28
504 (21 days)	1397 ± 27
672 (28 days)	1506 ± 32

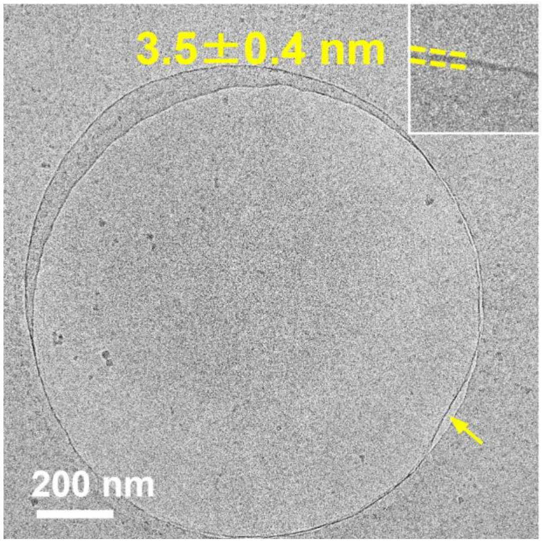

Fig. S10 Cryo-TEM image of **bola-triEGSQ** vesicle at 14 days post-mixing (diameter: approx. 1200 nm).

References for SI

S1 Y. Nishizaki, N. Sugimoto, T. Miura, K. Asakura, T. Suematsu, S. Korhonen, J. Lehtivarjo, M. Niemitz, G. Pauli, Quantum Mechanical Quantitative Nuclear Magnetic Resonance Enables Digital Reference Standards at All Magnetic Fields and Enhances qNMR Sustainability, *Anal. Chem.*, 2024, **96**, 9790−9798.

S2 D. A. Van Hal, J. A. Bouwstra, A. Van Resen, E. Jeremiasse, T. De Vringer, H. E. Junginger, Preparation and Characterization of Nonionic Surfactant Vesicles, *J. Colloid Interface Sci.*, 1996, **178**, 263–273.

S3 E. Van Tamelen, T. Curphey, The Selective in Vitro Oxidation of The Terminal Double Bonds in Squalene, *Tetrahedron Letters*, 1962, **3**, 121−124.

S4 T. Spencer, The Squalene Dioxide Pathway of Steroid Biosynthesis, *Acc. Chem. Res.*, 1994, **27**, 83−90.
